# A three-year multifaceted intervention to prevent obesity in children of Mexican-heritage

**DOI:** 10.1186/s12889-019-6897-8

**Published:** 2019-05-16

**Authors:** Banafsheh Sadeghi, Lucia L. Kaiser, Meagan M. Hanbury, Iraklis Erik Tseregounis, Ulfat Shaikh, Rosa Gomez-Camacho, Rex C. Y. Cheung, Alberto L. Aguilera, Linda Whent, Adela de la Torre

**Affiliations:** 1 Department of Internal Medicine, Division of General Medicine, School of Medicine, Patient Support Services Building, 4150 V Street, Suite 2400, UC Davis Medical Center, Sacramento, CA 95817 USA; 2 Department of Nutrition, University of California, Davis, One Shields Avenue, Davis, CA 95616 USA; 3 Center for Transnational Health, University of California, Davis, 328 D Street, Davis, CA 95616 USA; 4 Department of Epidemiology, University of California, Davis, One Shields Avenue, Davis, CA 95616 USA; 5 Department of Pediatrics, School of Medicine, Glassrock Building, 2521 Stockton Boulevard, Suite 2200, Sacramento, CA 95817 USA; 6 Office of Planning & Institutional Performance, Florida Gulf Coast University, 10501 FGCU Boulevard South, AB5- Suite 313, Fort Meyers, FL 33965 USA; 7 Decision Sciences Department, College of Business, San Francisco State University, 1600 Holloway Avenue, San Francisco, CA 94132 USA; 8 Office of the President, San Diego State University, 5500 Campanile Drive, San Diego, CA 92182-8000 USA

**Keywords:** Childhood obesity, Mexican-origin communities, Multifaceted, community-based intervention, Rural area communities

## Abstract

**Background:**

Obesity and overweight have increased dramatically in the United States over the last decades. The complexity of interrelated causal factors that result in obesity needs to be addressed within the cultural dynamic of sub-populations. In this study, we sought to estimate the effects of a multifaceted, community-based intervention on body mass index (BMI) among Mexican-heritage children.

**Methods:**

*Niños Sanos, Familia Sana* (Healthy Children, Healthy Family) was a quasi-experimental intervention study designed to reduce the rate of BMI growth among Mexican-heritage children in California’s Central Valley. Two rural communities were matched based on demographic and environmental characteristics and were assigned as the intervention or comparison community. The three-year intervention included parent workshops on nutrition and physical activity; school-based nutrition lessons and enhanced physical education program for children; and a monthly voucher for fruits and vegetables. Eligible children were between 3 and 8 years old at baseline. Intent-to-treat analyses were estimated using linear mixed-effect models with random intercepts. We ran a series of models for each gender where predictors were fixed except interactions between age groups and obesity status at baseline with intervention to determine the magnitude of impact on BMI.

**Results:**

At baseline, mean (SD) BMI z-score (zBMI) was 0.97 (0.98) in the intervention group (*n* = 387) and 0.98 (1.02) in the comparison group (*n* = 313) (NS). The intervention was significantly associated with log-transformed BMI (β = 0.04 (0.02), *P* = 0.03) and zBMI (β = 0.25 (0.12), *P* = 0.04) among boys and log-transformed BMI among obese girls (β = − 0.04 (0.02), P = 0.04). The intervention was significantly and inversely associated with BMI in obese boys and girls across all age groups and normal weight boys in the oldest group (over 6 years) relative to their counterparts in the comparison community.

**Conclusions:**

A community-based, multifaceted intervention was effective at slowing the rate of BMI growth among Mexican-heritage children. Our findings suggest that practitioners should consider strategies that address gender disparities and work with a variety of stakeholders to target childhood obesity.

**Trial registration:**

clinicaltrials.gov Identifier: NCT01900613. Registered 16th July 2013.

## Background

Pediatric obesity and overweight prevalence has more than tripled from 1971 to 2011 [1], triggering health problems including high blood pressure, type 2 diabetes, and elevated blood cholesterol levels [2]. Common co-morbidities include low self-esteem, negative body image, and depression. According to the American Heart Association, these health issues are occurring earlier in life and are linked to an accelerated risk of obesity-related disease and earlier death in adulthood [3–5]. The incremental health care costs of pediatric obesity are estimated at $14 billion [6].

Two-thirds of Latinos living in the US are Mexican-heritage. At 17.8% of the total population, as of 2016, the Latino community is the majority minority population in the US [7]. Most recent data (2011–2014) indicate 22.4% of Hispanic males and 21.4% of Hispanic females ages 2–19 in the US are obese compared with 14.3% of Non-Hispanic white males and 15.1% of Non-Hispanic white females in the same age category [8]. Additionally, a recent study from Northern California found that 18.2% of Hispanic boys and 15.2% of Hispanic girls, ages 3–5 years, were obese [9]. Preventive strategies targeting Mexican-American children at risk of obesity are critical. An increasing number of community-based interventions report body mass index (BMI) outcomes in Latino children [10–15]. Study characteristics associated with improved BMI outcomes include longer duration and/or greater intensity of intervention, targeting parenting skills, having dedicated staff, and utilizing behavior-change theories [11]. Noticeably missing in the literature, however, are long-term, community-based interventions addressing the economic and cultural factors in predominantly rural Mexican origin communities at high risk of childhood obesity. High rates of poverty, food insecurity, and social isolation in these communities create challenges that may require long-term economic and educational components to reverse childhood obesity trends [16, 17]. Successful interventions may include culturally nuanced education targeted towards both caregivers and children combined with economic supports to purchase healthy foods. Furthermore, it is important that interventions focus on all children, as normal and overweight children are at still at risk of becoming obese.

*Niños Sanos, Familia Sana* (NSFS - Healthy Children, Healthy Family) measured the impact of a multifaceted, community-based intervention on children’s BMI in an underserved, rural Mexican-heritage community in California’s Central Valley. The intervention and comparison communities in this study match residential environment and demographics of the majority of California’s agricultural workforce [18]. Gender differences in response to the intervention were examined because national data [19] for Mexican-American adolescents indicate higher prevalence of overweight and obesity among boys (27.9%) compared to girls (18.0%). Given the relatively long duration of the intervention, age group at the start of the intervention was also expected to influence growth trajectory. This goal of the intervention was to reduce the rate of BMI growth among participating children and this paper addresses the changes in anthropometric measures that occurred from baseline to the end of the three-year intervention.

## Methods

### Design

NSFS was a quasi-experimental, community-based, multifaceted intervention study designed to slow BMI growth among Mexican-heritage children. Two rural communities were matched based on demographic and environmental characteristics and assigned as the intervention or comparison community through a coin toss. Behavioral interventions included parent classes on nutrition and physical activity; school-based nutrition and enhanced physical education for children; and a monthly voucher for participant families to purchase fruits and vegetables. The methodology and study protocol have been described elsewhere [20].

Recruitment and enrollment occurred between August 2011 and September 2014, data collection between March 2012 and April 2016, and intervention phase from September 2012 through August 2015. Participants were added to the study on an on-going basis and completed baseline surveys and anthropometric measurements prior to beginning interventions, regardless of enrollment date.

The University of California (UC), Davis Institutional Review Board approved this study. Legal guardians of participant children provided written informed consent.

### Sample

The intervention and comparison communities were located in California’s rural Central Valley. These towns had over 80% Mexican-heritage populations and an agricultural employment base. Additional criteria for community selection included no existing comprehensive nutrition intervention and agreement by community/school district leaders to work with researchers.

To be eligible, children must have: (1) been between 3 and 8 years old at baseline (born between December 3, 2004 and October 1, 2009, or in 2nd grade or lower during the 2012–2013 school year); (2) had at least one Mexican-heritage parent; and (3) resided in a participating school district. Children who moved out of the school district, were no longer living at home, or had parents no longer interested in participating, were dropped from the study. Excluded from the analysis were underweight children, children with no measurement data, and children with a chronic condition (including: Down’s syndrome, leukemia, anemia, or a limiting handicap).

Based on an estimation of the number of children enrolled in the targeted school districts, we anticipated that our sample would contain approximately 400 children in each group. This sample size was deemed sufficient to detect an effect difference between groups in the mean change in standardized BMI (z-scores) of as small as 0.20 standard deviations with 80% power.

### Interventions

The nutrition intervention included education delivered to parents at monthly “family nights” and to children in the school setting. The process of culturally tailoring key obesity prevention messages, recommended by the American Academy of Pediatrics, and delivering lessons based on the Social Learning Theory, has been previously described [21]. A University of California Cooperative Extension (UCCE) nutrition specialist trained a bilingual local nutrition educator to deliver the family night curriculum. Each class lasted about 1 hour and included a discussion, hands-on activity, and food demonstration. To accommodate family schedules, small-group sessions were offered to 15 or fewer parents several mornings and evenings each month.

Twenty-two different classes were offered, including an orientation, and a healthy food cook-off event. Class topics and materials are available on the UCCE website [22]. Based on class attendance logs, 49% of parents attended five or more classes; 21% attended no classes. Each month, the same topic was covered at all classes. Up to 16 time slots were offered on a monthly basis. Though the specific topic varied on a monthly basis (for example, Shopping, Healthy Snacks, Walking), the classes reinforced the same key obesity prevention messages as recommended in reference 21 (for example, eat more fruit and vegetables, reduce sugar-sweetened beverages, increase active play). Since recruitment was on-going, monthly family nights commenced before all families started the program. Classes were offered from September 2012 through February 2015, skipping summer months when most families engaged in agricultural work and offering fewer in December when many families went to Mexico for Christmas. From February 2015 to June 2015, reinforcement activities (hands-on cooking classes) were offered since all the planned topics had been delivered.

UCCE nutrition educators and classroom teachers co-delivered science-based nutrition curricula, aligned with the California state standards, to children in preschool and grades K-3 in the intervention community [23]. Based on teacher-reported number of lessons delivered, 65% of students received seven or more lessons, not including food tasting activities. UCCE and the Fresno County Department of Public Health agreed to delay starting new nutrition and/or physical activity interventions in the intervention and comparison communities through the end of NSFS. Nevertheless, there were 13 teachers in the comparison community, compared to 56 teachers in the intervention community, who delivered nutrition lessons to their students during the 3 years of the intervention. Furthermore, we met quarterly with community leaders over the three-year period to maintain communication about health promotion activities (such as local health fairs). While such activities did occur in both communities, none of these could be considered more than usual public health outreach efforts.

The physical activity component delivered school-based physical education (PE). Researchers implemented the *Sport, Play and Active Recreation for Kids* (SPARK) *K-2 and Early Childhood (EC)* PE curricula in grades K-2 and preschools, respectively. A PE teacher delivered SPARK curriculum in 20–30-min exercise sessions with elementary school classes on a weekly basis. The PE teacher also trained classroom teachers, who incorporated SPARK activities into lesson plans. A research team member received SPARK EC training and helped preschool teachers incorporate the curriculum into the early childhood environment. The SPARK EC curriculum used in this study included a health–fitness focus and activities that improved child motor/sport skills. Lessons also integrated early childhood educational activities, including colors, shapes, animals, singing and vocabulary.

The economic component provided a monthly $25 fruit and vegetable voucher that allowed the same foods approved for the California Special Supplemental Nutrition Program for Women, Infants and Children Cash Value Voucher [24]. Funds were delivered via an electronic debit card, valid at the primary grocery store in the intervention community. Unused funds expired at the end of the month and the card was automatically reloaded. Over the course of the intervention, households spent 70% of allocated funds.

The parent education and voucher components began in September of 2012. After completing baseline data collection, families attended a voucher orientation, received their cards, and began attending the parent nutrition education classes. The school-based components were rolled-out after September 2012. Due to ongoing enrollment and participant choice, not all participants received the same dose of intervention.

### Data

Researchers collected anthropometric measurements from children at baseline and every 6 months thereafter for the next 4 years. Maternal anthropometric measurements were collected at baseline. Measures presented here include height, weight, and waist circumference. Research staff measured and weighed children and mothers in accordance with the Anthropometric Standardization Reference Manual [25]. Before each measurement period, data collectors received a 3-h training and were standardized using the technical error of measurement [26]. Maternal and child measurements were collected via the same procedures.

A digital scale (Model 874, Seca GmbH & Co. KG, Hamburg, Germany) was used to weigh participants to the nearest 0.1 kg. A portable stadiometer (Seca Model 213) was used to measure height to the nearest 0.1 cm. Abdominal circumference was measured with a body circumference measuring tape (QM2000 QuickMedical Corporate, Issaquah, WA, USA).

Bilingual research staff collected additional data on household size and income, child age and gender, and maternal age, education, ethnicity, and country of origin. Acculturation was measured using the Brief Acculturation Rating Scale for Mexican Americans [27]. Parents or legal guardians of eligible children were verbally administered survey instruments. The majority of respondents were mothers.

### Measures

#### Outcome variables

Child BMI, age- and sex-specific percentiles, and BMI z-scores (zBMI) were calculated using the Centers for Disease Control and Prevention (CDC) reference [28]. Statistical software (SAS, version 9.3, SAS Institute, Cary, NC) code was used to compute zBMI.

However, using zBMI from CDC charts for longitudinal analyses raises important concerns, including the suitability of using a cross-sectional referent for longitudinal purposes and longitudinal use of z-scores from transformations of non-Gaussian distributions [29]. Furthermore, growth-chart data beyond the 97th percentile are insufficient [30]. Therefore, in this paper, we analyzed both the logarithmic transformation of raw BMI (log BMI) and zBMI and included both the zBMI and log BMI results for comparability purposes with other studies. Waist circumference-to-height ratio (WCHTR) served as an additional measure of adiposity [31].

#### Explanatory variables

*Intervention* is a categorical variable with level 0 for individuals with no intervention and level 1 otherwise.

*Duration* measures the length of time the individual received the intervention. Duration spanned the date of enrollment or intervention start (whichever occurred last) through the date of the final anthropometric measurement or exiting the study (whichever occurred first). The maximum duration, 941 days, included individuals who enrolled prior to September 1, 2012 and remained enrolled through March 31, 2015.

*Days* measures the time between the start of intervention (September 1, 2012) and the date of anthropometric measurement. Baseline measurements that occurred before September 1st, 2012 were assigned a value of zero, as opposed to a negative value.

*Obesity* is a categorical variable defining the child’s weight status at first measurement. The levels were defined as: 1 = “normal” if BMI > 5th percentile and BMI <85th percentile; 2 = “overweight” if BMI ≥85th percentile and < 95th percentile; and 3 = “obese” if BMI ≥95th percentile [32].

*Age group* categorizes children into three classifications based on age during the first measurement period, irrespective of entry date: 1 = < 4.6 years at baseline; 2 = 4.6–6 years; and 3= > 6 years. The cut-points created equal sized groups, increasing intergroup comparability.

### Statistical analysis

We conducted intent-to-treat analyses using the statistical software R (version 3.2.2) (R Core Team, 2015), and models were fitted using the *lme* function in the nlme package [33]. Descriptive analyses detected differences between the intervention and comparison communities by comparing means and percentages with chi-square or student t-tests. We used a linear mixed-effect model with random intercept (adjusting for repeated measures for individuals) to assess changes in anthropometric measures. The analysis uses the full sample of measurement data and includes observations from children who lack a full set of measurements over each time period. Therefore, the analytical sample is unbalanced over time.

Gender differences of BMI means and trajectories and other anthropometric measures necessitated estimating sex-specific models. Primary outcomes were log BMI and zBMI. WCHTR was also used as an outcome variable. Each model included the explanatory variables listed above, interactions of intervention and obesity, and intervention and age group.

In models not presented, we separately added acculturation, mother’s birth place, mother’s education, and mother’s age to adjust for differences between the two communities at baseline. Due to high collinearity in these variables, we chose models that included birth place as our preferred specifications because those models retained the largest sample size.

## Results

**Fig. 1:**
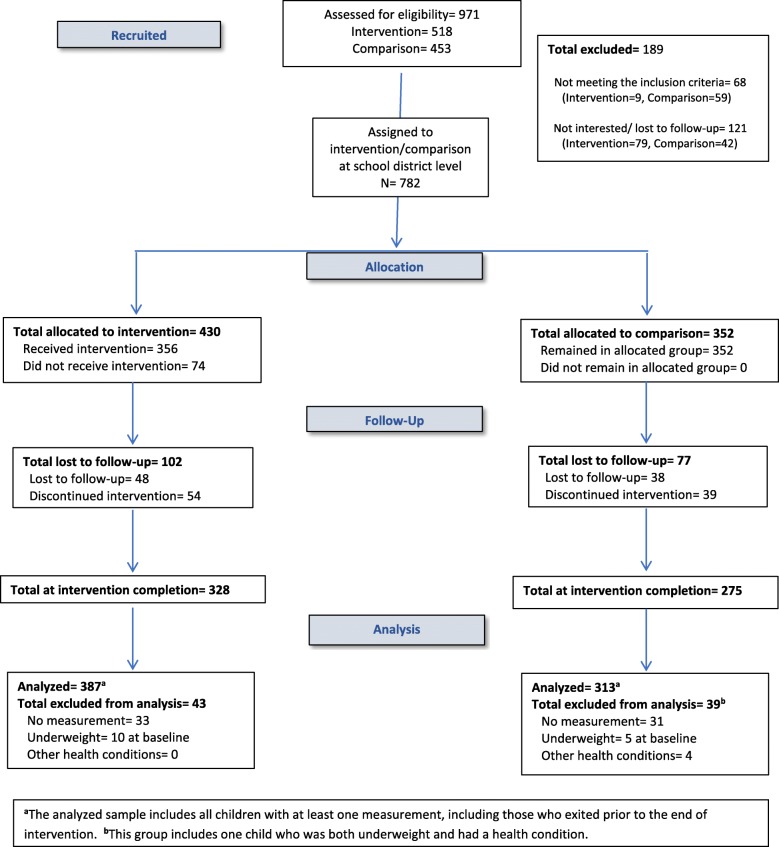
Consort Flow Diagram

Figure 1 displays the flow of participating children across the intervention and comparison groups. Throughout the study, 782 children were eligible and had parental/guardian consent, including 430 in the intervention group and 352 in the comparison group. Retention rates for the intervention-group and comparison-group were 76.0% (*n* = 328) and 78.1% (*n* = 275), respectively. The analytical sample included children with at least one set of anthropometric measurements at any point during the study, including those who exited before study completion: 387 and 313 in the intervention and comparison groups, respectively. Children who exited had younger and less acculturated mothers than children who completed the study. There was no difference in zBMI or BMI percentile between those who exited and remained (data not shown).

Table 1 shows baseline characteristics. Comparison-community mothers were significantly younger, less educated, and more likely to have been born in Mexico than intervention-community mothers. The multivariate analysis controlled for these variables. Otherwise, differences were not statistically significant between communities in any other baseline characteristics.

**Table 1:** Baseline Characteristics of Participant Children in the NSFS Study

Characteristics	Study Site			
	All (*N* = 700)	Intervention (n = 387)	Comparison (n = 313)	*P* Value^a^
Child				
Age, mean (SD), yrs	5.98 (1.31)	6.02 (1.34)	5.93 (1.28)	0.42
Sex				
Female, n (%)	355 (50.7)	182 (47.0)	173 (55.3)	0.11
Male, n (%)	345 (49.3)	205 (53.0)	140 (44.7)	0.13
Height, mean (SD), cm	110.40 (9.59)	110.47 (9.74)	110.27 (9.34)	0.81
Weight, mean (SD), kg	21.51 (5.53)	21.41 (5.29)	21.69 (5.94)	0.56
Waist circumference, mean (SD), cm	56.18 (7.16)	55.92 (6.79)	56.62 (7.75)	0.26
WCHTR, mean (SD)	0.51 (0.05)	0.51 (0.05)	0.51 (0.06)	0.10
BMI, mean (SD)	17.47 (2.46)	17.39 (2.21)	17.61 (2.85)	0.30
zBMI, mean (SD)	0.97 (0.99)	0.97 (0.98)	0.98 (1.02)	0.86
Obesity status at baseline				
Normal, n (%)	383 (54.7)	211 (54.5)	172 (55.0)	0.92
Overweight, n (%)	130 (18.6)	84 (21.7)	46 (14.7)	0.32
Obese, n (%)	187 (26.7)	92 (23.8)	95 (30.3)	0.77
Parent and Household				
Mother age, mean (SD), yrs	32.65 (6.80)	33.03 (6.98)	32.17 (6.54)	0.10
Mother BMI at baseline, mean (SD)	31.09 (6.53)	30.92 (6.09)	31.36 (7.21)	0.54
Mother education, mean (SD), yrs	9.49 (3.78)	9.85 (4.06)	9.04 (3.36)	0.006
Mother place of birth				
United States, n (%)	126 (18.6)	88 (23.4)	38 (12.6)	0.17
Mexico, n (%)	524 (77.4)	267 (71.0)	257 (85.4)	0.0001
Other, n (%)	27 (4.0)	21 (5.6)	6 (2.0)	0.72
Household size, mean (SD)	5.05 (1.46)	5.02 (1.51)	5.07 (1.41)	0.67
Household annual income, mean (SD), $	1859.71 (1072.66)	1919.96 (1096.27)	1783.83 (1039.24)	0.12
Family acculturation level				
Traditional, n (%)	423 (75.1)	212 (71.4)	211 (79.3)	0.06
Low bicultural, n (%)	69 (12.3)	38 (12.8)	31 (11.7)	0.91
High bicultural, n (%)	71 (12.6)	47 (15.8)	24 (9.0)	0.43
Poverty Status^b^				
Above Poverty Line, n (%)	288 (41.1)	162 (41.9)	126 (40.2)	0.77
Below Poverty Line, n (%)	412 (58.9)	225 (58.1)	187 (59.7)	0.74

Abbreviations: BMI, body mass index; zBMI, standardized body mass index; WCHTR, waist circumference-to-height ratio. ^a^*P* Value determined by Chi-square and Student’s t-test. ^b^Poverty Line determined from U.S. Department of Health and Human Services Federal Poverty Line guidelines (https://aspe.hhs.gov/2012-hhs-poverty-guidelines)

Table 2 presents regression results with log-BMI and zBMI as outcomes. After controlling for age group, mother’s birth place, and obesity status at the time of enrollment; time-interval from baseline to measurement (days); and participation duration, the intervention group (1 = treatment; 2 = comparison) was significantly associated with both log BMI (β = 0.04 (0.02), *P* = 0.03) and zBMI (β = 0.25 (0.12), *P* = 0.04) among boys. Among girls, the intervention term was only significant for children who were obese at baseline (ref normal wt status) (β = − 0.04 (0.02), P = 0.04). To examine the magnitude of the intervention’s effect on BMI, we ran a series of models for each gender with all the predictors fixed except interactions between age group and obesity status at baseline with the intervention. The intervention was significantly and inversely associated with BMI in obese boys in all age groups and normal weight boys in the oldest age group (over 6 years). The intervention resulted in significantly lower BMI in obese girls in all three age groups relative to their counterparts in the comparison group.

**Table 2:** Regression Results of log-BMI and zBMI

		log-BMI		zBMI	
		Girls (*n* = 347)	Boys (*n* = 330)	Girls (*n* = 347)	Boys (n = 330)
Predictors	N (# Observation)	1295	1236	1295	1236
Intercept	Β	2.70	2.69	0.30	0.14
	Standard error	0.02	0.02	0.12	0.15
	*P* value	<0.001	<0.001	0.01	0.36
Intervention	Β	0.01	0.04	−0.02	0.25
1 = treatment; 2 = comparison	Standard error	0.02	0.02	0.10	0.12
	*P* value	0.69	0.03	0.81	0.04
Duration of being in the program	Β	0.00	0.00	0.00	0.00
	Standard error	0.00	0.00	0.00	0.00
	*P* value	0.83	0.78	0.74	0.43
Days from the start of intervention to each measurement	Β	0.00	0.00	0.00	0.00
	Standard error	0.00	0.00	0.00	0.00
	*P* value	<0.001	<0.001	0.13	0.22
Overweight at baseline (ref. normal)	Β	0.12	0.11	0.94	0.96
	Standard error	0.02	0.02	0.11	0.14
	*P* value	<0.001	<0.001	<0.001	<0.001
Obese at baseline (ref. normal)	Β	0.32	0.32	1.82	1.92
	Standard error	0.01	0.02	0.09	0.11
	*P* value	<0.001	<0.001	<0.001	<0.001
Age group 2 (ref. age group 1)	Β	0.04	0.04	0.05	0.02
	Standard error	0.02	0.02	0.09	0.12
	*P* value	0.01	0.05	0.58	0.88
Age group 3 (ref. age group 1)	Β	0.11	0.10	0.11	0.14
	Standard error	0.02	0.02	0.10	0.11
	*P* value	<0.001	<0.001	0.25	0.23
Mother’s place of birth^**a**^ 1 = United States; 2 = Mexico	Β	0.01	−0.01	0.09	−0.04
	Standard error	0.01	0.01	0.07	0.08
	*P* value	0.21	0.44	0.19	0.67
Intervention × age group 2	Β	0.00	−0.03	− 0.06	− 0.22
	Standard error	0.02	0.02	0.13	0.15
	*P* value	0.91	0.16	0.63	0.16
Intervention × age group 3	Β	−0.03	−0.05	−0.05	− 0.30
	Standard error	0.02	0.02	0.13	0.15
	*P* value	0.25	0.04	0.73	0.04
Intervention × overweight at baseline	Β	0.02	0.01	0.17	−0.05
	Standard error	0.02	0.03	0.15	0.17
	*P* value	0.51	0.79	0.26	0.78
Intervention × obese at baseline	Β	−0.04	−0.06	−0.05	− 0.08
	Standard error	0.02	0.02	0.13	0.14
	*P* value	0.04	0.01	0.73	0.56

Abbreviation: *BMI* body mass index, *zBMI* standardized body mass index. ^**a**^Households that were not of Mexican-heritage did not meet eligibility criteria

**Fig. 2:**
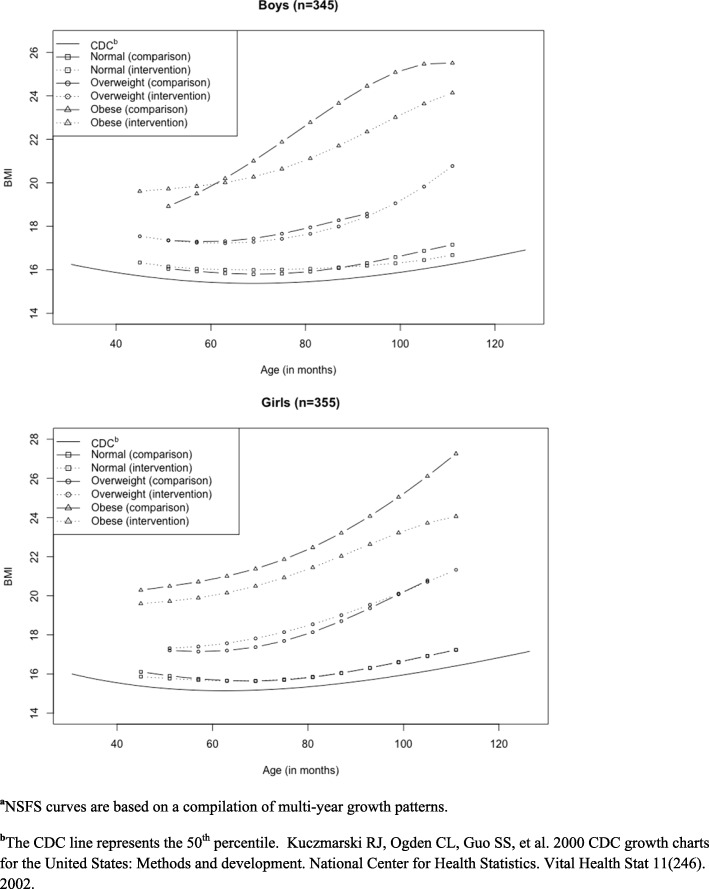
NSFS BMI Trajectories^**a**^ by Baseline Weight Status, by Gender

Figure 2 presents the CDC expected BMI (50th percentile by age in months) along with NSFS participants’ observed BMI, stratified by community, gender, and obesity status at baseline. In boys across all three categories of baseline obesity status, BMI in the comparison group is higher than the intervention group at the older end of the age spectrum. Overall, obese girls in the comparison community have higher BMI relative to the intervention site, however the gap widens among older girls.

Table 3 presents regression results with WCHTR as the outcome. The results for boys’ WCHTR were similar to BMI. The intervention term was insignificant in models of girls’ WCHTR.

**Table 3:** Regression Results of Waist Circumference-to-Height Ratio

		Girls (*n* = 347)	Boys (*n* = 329)
Predictors	N (# Observation)	1280	1220
Intercept	Β	0.48	0.47
	Standard error	0.01	0.01
	*P* value	< 0.001	< 0.001
Intervention	Β	0.00	0.02
1 = treatment; 2 = comparison	Standard error	0.01	0.01
	*P* value	0.56	0.02
Duration of being in the program	Β	0.00	0.00
	Standard error	0.00	0.00
	*P* value	0.95	0.81
Days from the start of intervention to each measurement	Β	0.00	0.00
	Standard error	0.00	0.00
	*P* value	< 0.001	< 0.001
Overweight at baseline (ref. normal)	Β	0.04	0.03
	Standard error	0.01	0.01
	*P* value	< 0.001	0.001
Obese at baseline (ref. normal)	Β	0.11	0.11
	Standard error	0.01	0.01
	*P* value	< 0.001	< 0.001
Age group 2 (ref. age group 1)	Β	0.00	0.00
	Standard error	0.01	0.01
	*P* value	0.88	0.87
Age group 3 (ref. age group 1)	Β	0.00	0.01
	Standard error	0.01	0.01
	*P* value	0.49	0.49
Mother’s place of birth^**a**^ 1 = United States; 2 = Mexico	Β	0.01	0.00
	Standard error	0.00	0.01
	*P* value	0.02	0.70
Intervention × age group 2	Β	0.00	−0.01
	Standard error	0.01	0.01
	*P* value	0.84	0.23
Intervention × age group 3	Β	−0.01	−0.02
	Standard error	0.01	0.01
	*P* value	0.54	0.04
Intervention × overweight at baseline	Β	0.01	0.00
	Standard error	0.01	0.01
	*P* value	0.40	0.86
Intervention × obese at baseline	Β	−0.01	−0.03
	Standard error	0.01	0.01
	*P* value	0.33	0.004

^**a**^Households that were not of Mexican-heritage did not meet eligibility criteria

Exploration of the three-way interaction of age group, intervention and baseline obesity status yielded insignificant associations of terms with outcomes (data not shown).

## Discussion

The multifaceted intervention in this study reduced BMI trajectory in obese boys and girls, across all age groups, in the intervention community relative to the comparison community. The intervention was also associated with lower BMI trajectories for normal weight boys and girls in the oldest age category (over 6 years), however this difference was only significant for boys. WCHTR, another measure of adiposity, was significantly and inversely associated with the intervention for obese boys and older boys in all weight categories.

These findings are similar to those reported by several studies targeting Latino children. Like NSFS, Barkin et al. reported obese children to have the largest reduction in BMI in response to their intervention [12]. Both studies targeted Latino children, starting at ages 2–6 years, through culturally-adapted educational interventions focusing on nutrition, physical activity, and parenting skills. In a non-randomized study targeting Mexican-American children in Headstart centers, Yin et al. observed more favorable pre-post changes in weight z-score in the group receiving an 18-week center- and home-based intervention, compared to a nonintervention group [34]. Haines et al. [35] randomized 121 low-income, predominantly Hispanic, families to a six-month educational intervention. Themes were similar to those presented in NSFS and focused on household routines, including family meals, sufficient sleep time, and limits on TV time. Intervention children showed significant decreases in BMI. However, Alexander et al. [15] did not find that a school-based, multifaceted intervention targeting first and second graders decelerated BMI gains over a six-month follow-up period among the most obese children, though improvements were observed in overweight children.

Most interventions targeting this population have been considerably shorter in duration than NSFS [12, 13, 34, 35]. However, *Aventuras Para Niños* (Adventures for Children) [13] was a three-year intervention with a factorial design that examined the effect of family- and/or community-level interventions on BMI and behavioral outcomes. Although significant changes in parenting behaviors occurred, no improvements in children’s zBMI or BMI percentiles were observed, either in the total sample or by gender and initial BMI status. That study did not report on differences in BMI trajectories. Important differences that may have influenced outcomes include location of site (NSFS, rural vs. *Aventuras*, urban); provision of an economic incentive in NSFS (no incentive in Aventuras); and younger age of children at baseline in NSFS (3–8 years) compared to *Aventuras* (K-1st grade).

Similar to the studies cited above [12, 13, 15, 34, 35], NSFS did not result in a significant effect on zBMI in girls. Instead, log BMI as an outcome revealed changes that might be expected in sample subgroups. Other researchers have noted that zBMI may not be the optimal measure for assessing adiposity change over time in young children and adolescents [36]. Both log BMI and zBMI models show the significant effect of intervention in the older children group. However, only the log BMI model detects a significant impact of intervention on children with obesity at the baseline. We believe the reasons that the zBMI model fails to detect this association are twofold. First, according to the CDC the standard growth charts do not accurately estimate very high BMIs. In our sample, 26.7% of children have BMI over 95th percentile. Second, in longitudinal and intervention studies the changes in L (normality) and S (dispersion) parameters are particularly large in very high BMI children and cause even more instability in zBMI [29, 30, 37].

In long-term, community-based obesity prevention studies that include all healthy children with normal, overweight, or obese weight status at baseline, it is not surprising to find certain subgroups responding more strongly than others to the intervention. In a review of interventions among Latino children, Branscum and Sharma noted that the more successful interventions had targeted children at higher risk (more overweight and obese) with more room for improvement [11]. An intervention’s potential to impact growth trajectories might also be expected to differ by age; more success has been documented in children 6–11 years, compared to younger or older children [38]. Gender differences also can influence response to an intervention. Growth trajectories for sons of Mexican-American immigrant mothers begin to increase sharply around 4.5 years, a pattern not observed in girls [39]. This gender effect may explain why an interaction between the intervention and older age (over 6 years) was observed in boys but not girls. NSFS researchers previously reported that the rate of BMI growth for obese boys slowed significantly after the first intervention year [40], whereas the response for obese girls was delayed until the third year. In Mexico, a preference for sons, coupled with indulgent feeding of boys, has been attributed to heavier body weights [41]. Messages in NSFS classes discouraged indulgent feeding, which may have had a differential impact on boys. Furthermore, Latino parents tend to become concerned when their children become obese, as opposed to overweight, and apply different standards to boys and girls [42]. Therefore, parents may be slower to recognize or respond to a problem in daughters than sons, suggesting value to gender-nuanced strategies within this population.

This study has some limitations. The intent-to-treat analysis assumes intervention compliance and does not consider the dose-response of individual components in the intervention package. For example, not all parents attended the same number of nutrition education workshops. Furthermore, the study population is homogenous and culturally unique, and results may not be generalizable to a more diverse set of children. The sample homogeneity may restrict the ability to examine covariates known to be correlated with child weight, including acculturation and mother’s education and age. Finally, a cluster-randomized design, including more than two communities per treatment, would have been a stronger design, however was not feasible due to scope and funding.

This study also has a number of strengths. This is one of the first long-term studies to focus on young Mexican-heritage children in a rural setting. The study benefited from broad participation of a transdisciplinary research team of doctors, nutritionists, economists, and behavioral scientists who collaborated with community members and school district leaders to design and implement an intervention that addressed both knowledge gaps and socioeconomic barriers to maintaining a healthy weight.

## Conclusions

A community-based, multifaceted intervention was effective in slowing the BMI growth rate among obese Mexican-heritage children and normal weight boys older than 6 years old. In addition, girls and boys respond differently, implying gender specific strategies should be considered. The findings also suggest that to enhance effectiveness of interventions, clinicians should work with a variety of community-based stakeholders, including parents, school officials, and policy makers.

## Abbreviations

BMI: Body Mass Index
CDC: Centers for Disease Control and Prevention
EC: Early Childhood
NSFS: *Niños Sanos, Familia Sana*
PE: Physical Education
SPARK: Sport, Play and Active Recreation for Kids
UC: University of California
UCCE: University of California Cooperative Extension
USDA: US Department of Agriculture
WCHTR: Waist circumference-to-height ratio
WIC: Special Supplemental Nutrition Program for Women, Infants and Children
zBMI: BMI z-score
